# Why Blood Glucose Control Matters for the Kidney

**DOI:** 10.1371/journal.pmed.0020056

**Published:** 2005-02-22

One of the most common and most serious complications of both type 1 and type 2 diabetes is diabetic nephropathy. It occurs in around 30% of patients with type 1 diabetes and 10% to 40% of patients with type 2 diabetes. Diabetic nephropathy is the leading cause of renal failure in the developed world. The main effect of diabetic nephropathy is proteinuria, initially in very small amounts but which increases, leading to nephrotic syndrome and end-stage renal disease in most cases.[Fig pmed-0020056-g001]

**Figure pmed-0020056-g001:**
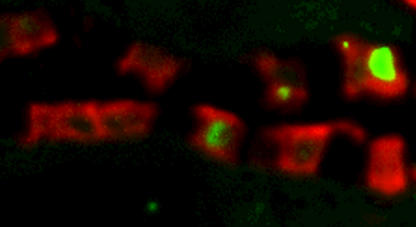
Apoptotic renal tubular cells in diabetic nephropathy

Various risk factors in individuals with diabetes are known to increase the chance of developing diabetic nephropathy, including South Asian or African background, male sex, long history of diabetes, poor blood sugar control, high blood pressure, and smoking. One early change associated with diabetic nephropathy is degeneration of the renal tubular epithelium, but the exact cause of this at the cellular level is unclear. Erwin Böttinger and colleagues have dissected out one key point in the progression to diabetic nephropathy. They looked at cell lines of renal tubular cells from humans and mice and kidney biopsies from patients with diabetic nephropathy, patients with non-diabetic renal disease, and mice with genetic and induced diabetes. In the human cell lines they showed that glucose induced the expression of CD36, a receptor known to have a role in adhesion and signal transduction (in addition to being the receptor for malaria-infected erythrocytes). They then went on to show that apoptosis of these cells occurred in the presence of glycated (glucose-modified) albumins or free fatty acids, which are present in increased amounts in patients with diabetes, and that CD36 was essential for the apoptosis to occur. They then examined how CD36 triggered apoptosis and found that it involved src kinase, p38 MAP kinase, and caspase 3.

Comparing mice and humans, the researchers found that the two species are not alike: diabetic mice did not show an increase in tubular expression of CD36—even though the gene is present in mice—and had normal tubular epithelium and no tubular apoptosis. They confirmed this difference between humans and mice by showing that normal mouse epithelial cell lines were resistant to apoptosis caused by the glycated albumins; however, artificially expressing CD36 in these lines made them susceptible to apoptosis by these modified albumins.

These results provide insight into one of the crucial steps in diabetic nephropathy and, in humans at least, might help to explain why high blood glucose is so damaging to the kidney, hence providing a good reason—if another is needed—for encouraging patients to control blood glucose as tightly as possible.

